# Recommendations for diagnosing STIC: a systematic review and meta-analysis

**DOI:** 10.1007/s00428-021-03244-w

**Published:** 2021-12-01

**Authors:** Joep M. A. Bogaerts, Miranda P. Steenbeek, Majke H. D. van Bommel, Johan Bulten, Jeroen A. W. M. van der Laak, Joanne A. de Hullu, Michiel Simons

**Affiliations:** 1 Department of Pathology, Radboud University Medical Center, Postbus 9101, 6500 HB, Nijmegen, The Netherlands; 2 Department of Obstetrics and Gynecology, Radboud University Medical Center, Nijmegen, The Netherlands; 3 Diagnostic Image Analysis Group, Radboud University Medical Center, Nijmegen, The Netherlands

**Keywords:** BRCA mutation, Fallopian tube, Ovarian carcinoma, Risk reducing salpingo-oophorectomy, Serous tubal intraepithelial carcinoma, STIC

## Abstract

Our understanding of the oncogenesis of high-grade serous cancer of the ovary and its precursor lesions, such as serous tubal intraepithelial carcinoma (STIC), has significantly increased over the last decades. Adequate and reproducible diagnosis of these precursor lesions is important. Diagnosing STIC can have prognostic consequences and is an absolute requirement for safely offering alternative risk reducing strategies, such as risk reducing salpingectomy with delayed oophorectomy. However, diagnosing STIC is a challenging task, possessing only moderate reproducibility. In this review and meta-analysis, we look at how pathologists come to a diagnosis of STIC. We performed a literature search identifying 39 studies on risk reducing salpingo-oophorectomy in women with a known BRCA1/2 PV, collectively reporting on 6833 patients. We found a pooled estimated proportion of STIC of 2.8% (95% CI, 2.0–3.7). We focused on reported grossing protocols, morphological criteria, level of pathologist training, and the use of immunohistochemistry. The most commonly mentioned morphological characteristics of STIC are (1) loss of cell polarity, (2) nuclear pleomorphism, (3) high nuclear to cytoplasmic ratio, (4) mitotic activity, (5) pseudostratification, and (6) prominent nucleoli. The difference in reported incidence of STIC between studies who totally embedded all specimens and those who did not was 3.2% (95% CI, 2.3–4.2) versus 1.7% (95% CI, 0.0–6.2) (*p* 0.24). We provide an overview of diagnostic features and present a framework for arriving at an adequate diagnosis, consisting of the use of the SEE-FIM grossing protocol, evaluation by a subspecialized gynecopathologist, rational use of immunohistochemical staining, and obtaining a second opinion from a colleague.

**Supplementary Information:**

The online version contains supplementary material available at 10.1007/s00428-021-03244-w.

## Introduction

Epithelial ovarian cancer (EOC) is the most lethal gynecological cancer. The most common histological subtype of EOC is high-grade serous carcinoma (HGSC), which is accountable for the majority of EOC deaths [1, 2]. HGSC has a poor prognosis, mainly because it is often diagnosed at an advanced stage. To date, no successful screening tools for early detection of HGSC have been found [3]. Therefore, women who are at an increased risk of developing HGSC are counselled on risk reducing salpingo-oophorectomy (RRSO). This group mainly consists of carriers of *BRCA1/2* pathogenic variant (PV), who have a life-time risk of 16–44% to develop EOC [4]. Though RRSO has negative side effects linked to the iatrogenically induced premature menopause, it has proven to be a very successful strategy in order to reduce HGSC risk by 80–96% [5, 6].

**Fig. 1:**
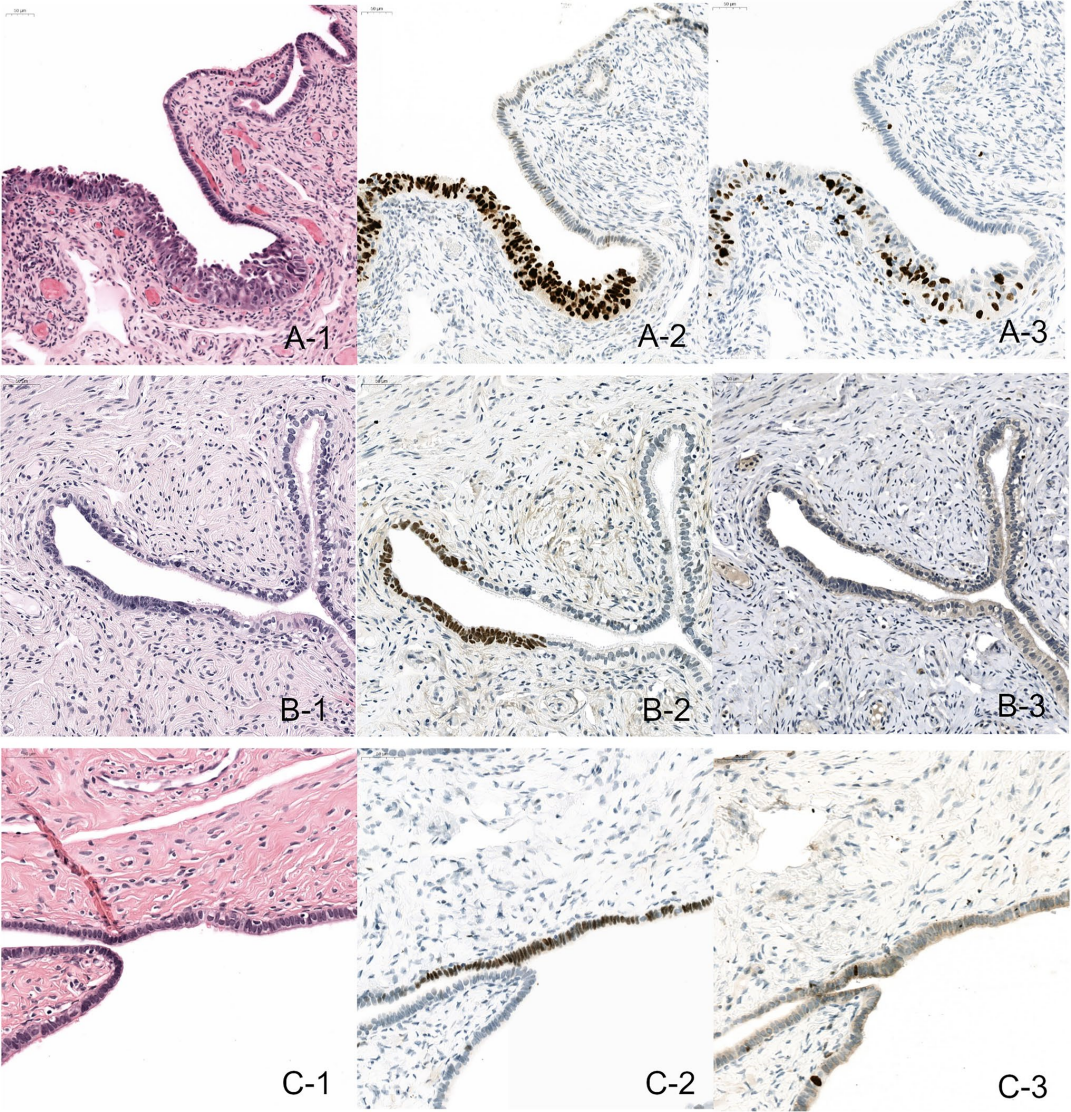
A: Example of a STIC, with H&E showing aberrant morphology (A-1), p53 overexpression (A-2), and a high Ki-67 labelling index (A-3). B: Example of a STIL, with H&E showing moderately aberrant morphology (B-1), p53 overexpression (B-2), and a low Ki-67 labelling index (B-3). C: Example of a P53 signature, with no aberrant morphology (C-1) p53 overexpression (C-2) and a low Ki-67 labelling index (C-3)

Our understanding of the pathogenesis of HGSC has greatly improved over the last few decades. Convincing evidence for a precursor lesion, originating in the fallopian tubes, has been found. This precursor lesion is most commonly referred to as serous tubal intraepithelial carcinoma (STIC). Correspondence between *TP53* mutations in STIC lesions and in concomitant HGSC provides evidence for a clonal relationship [7]. Moreover, an additional study on telomere length showed that STIC should be considered as a precursor lesion, rather than a non-invasive metastasis of carcinoma [8]. Other aberrant lesions in fallopian tube epithelium have also been identified such as serous tubal intraepithelial lesions (STIL) and p53 signatures. These lesions also contain *TP53* mutations, but lack the full cytomorphological and immunophenotypical features of STIC. Sometimes these lesions are grouped together as early serous proliferations (ESP). When these ESP present in an isolated status, no substantial malignant potential has been objectified so far [9]. Examples of STIC, STIL, and p53 signature lesions are shown in Fig. 1.

With the discovery that precursor lesions to HGSC originate in the fallopian tube, possible alternative preventive strategies for woman at an increased risk of developing HGSC emerge. Such strategies, consisting of a salpingectomy with delayed oophorectomy are currently being studied [10]. To also reduce ovarian cancer risk for low-risk women, an opportunistic salpingectomy can be considered under the appropriate circumstances. This means that the fallopian tubes are removed when a patient undergoes abdominal surgery for another benign indication [11, 12]. Thirteen FIGO (International Federation of Obstetrics and Gynecology) member societies currently have statements regarding opportunistic salpingectomy, whereby a majority support considering this practice [13].

STIC diagnosis is very rare for women undergoing salpingectomy for benign indications, (< 0.01%) [14]. In contrast, 11 to 61% of HGSC cases present with a concomitant STIC lesion [15]. Not all cases of HGSC have a clear precursor lesion, for which there may be several reasons. First of all, HGSC is often diagnosed in an advanced stage, whereby the fallopian tube can be obliterated or overgrown with carcinoma. Second, there may be sampling error, or STIC may remain unreported in the pathology report because it has no clinical consequences in the setting of HGSC. A third reason might be found in the “precursor escape” model, presented by Soong et al. They postulated a dual model, where next to STIC, other lesions, such as STIL or p53 signatures, might exfoliate precursor cells onto the ovaries or peritoneal cavity. These exfoliated cells could then, in a later stage, undergo malignant transformation, without leaving remnants of a precursor lesion in the fallopian tube itself [16]. Though the exact pathogenesis of HGSC is not yet fully unravelled, it is clear that the fallopian tube and especially STIC play an important role in it.

On the individual patient level, the detection of STIC is important, because it can have implications on prognosis. For example, in the case of RRSO, STIC is related to an increased risk of HGSC of the peritoneum [16, 17]. So far, it is not clear whether the identification of STIC should lead to additional staging and/or chemotherapy [18]. Additionally, reliable STIC diagnosis would be an absolute requirement in the setting of alternative risk reducing strategies. In these studies, the detection of STIC indicates an increased risk for HGSC and would prompt for an immediate oophorectomy [10]. Finally, on a population level, recognizing and adequately classifying STIC and other lesions, such as STIL and p53 signatures, are important in better understanding the oncogenesis of HGSC.

Multiple efforts to improve STIC detection have been initiated over the years. Grossing protocols, such as the “Sectioning and Extensively Examining the FIMbriated end” (SEE-FIM) have been developed [19]. Next to that, multiple diagnostic algorithms have been proposed on how to approach a STIC diagnosis, with the aim of assisting the pathologist towards a more consistent diagnosis.

Our objective is to set up a systematic review to provide an overview of current practices in the pathological diagnosis “STIC.” We will cover the use of grossing protocols, morphological criteria, training level of the pathologist, and the use of immunohistochemical stains (IHC). Because STIC and its diagnostic approach are commonly best described in studies on RRSO from *BRCA1/2*-PV carriers, we limited ourselves to these studies.

## Materials and methods

### Search strategy

A literature search strategy was designed for Embase, Medline, and Cochrane (CENTRAL) from inception until 1 September 2020*,* with search terms for *BRCA,* risk-reducing surgery, and pathological outcome. Three independent researchers (JBo/MSt/JHe) first screened the studies by title and abstract and secondly by full text. Each study was assessed by at least 2 researchers. Discrepancies were resolved by discussion or consultation of a third researcher (MSi). The review was performed in concordance with a protocol registered in PROSPERO (CRD42020120728). The search protocol is provided in the supplementary appendix.

### Study selection

All studies describing the pathology results of a risk-reducing salpingo-oophorectomy, performed among *BRCA1/2* PV carriers, aimed at defining the incidence or describing the histopathological characteristics of ovaries and fallopian tubes, were considered eligible for inclusion. Though STIC is also reported in women without a known *BRCA1/2* PV, the incidence in this group is low. As the incidence rates will be used to compare the effectiveness of various diagnostic features, this group might form a bias and was therefore excluded. For studies containing only summarized results for *BRCA1/2*-PV carriers, authors were asked to provide the subgroup data. Articles were excluded if these data remained unavailable. Articles written in another language than English or Dutch, conference abstracts, case reports, and review articles were also excluded.

### Data extraction

The primary outcomes recorded were incidence of STIC, morphological characteristics, the use of grossing protocols, the use and role of IHC, and whether a subspecialized gynecopathologist assessed the pathology specimens. Secondary outcomes included the incidence of invasive carcinomas hyperplasia, atypia, p53-signatures, and STIL. Sensitivity analyses were performed on the method by which tissue was embedded (not done/unknown, totally embedded, or in conformity with the SEE-FIM protocol), whether a subspecialized gynecological pathologist made the pathological assessment (yes/no/unknown) and on the use of IHC (yes/no/unknown). The authors were contacted in case of missing data or inconsistencies.

### Risk of bias within studies assessment

The methodological quality was independently assessed by two review authors (JBo/MSt) according to the standardized quality assessment tool for observational cohort and cross-sectional studies of the National Heart, Lung and Blood institute (NIH). Disagreements were resolved by discussion of consultation of a third review author (MSi).

### Statistical analysis

For the statistical analysis, with random effect models, and plots, we used R (*A language and environment for statistical computing; R Foundation for Statistical Computing, Vienna, Austria, version 4.0.4, packages “meta” and “dplyr”).*

## Results

### Study selection

The literature search identified 4133 studies. The selection of studies is displayed in the PRISMA flow diagram, provided in the supplementary appendix (supplement Fig. 1). After removal of 1976 duplicates, the remaining 2157 studies were screened. We excluded 1959 studies based on title and abstract, and another 159 studies were excluded after full-text assessment. A total of 39 studies met the inclusion criteria. A summary of the strengths and weaknesses of study quality is provided in the supplementary appendix (supplement Fig. 2).

### Study characteristics

**Fig. 2:**
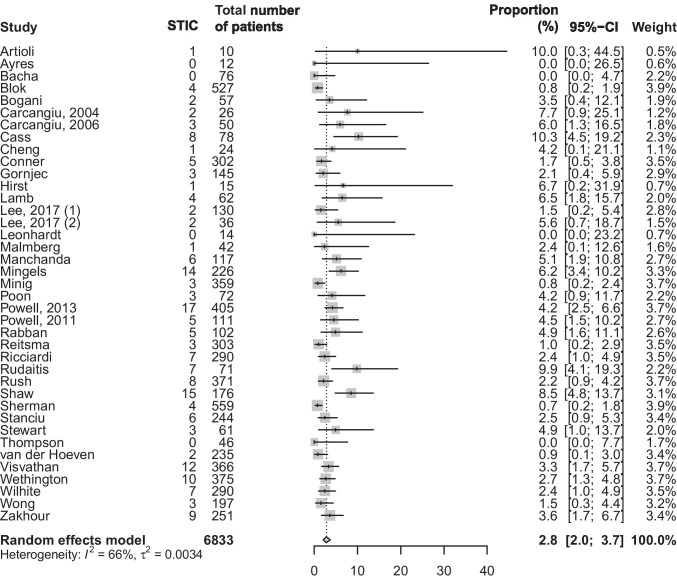
Forest plot on the proportion of STIC in included studies

The characteristics of all included studies are shown in Table 1. Studies were published between 2004 and 2020 and consisted of 10 prospective studies and 29 retrospective studies. The 39 studies included in this review collectively reported on 6833 patients, whereby 3642 patients carried a known *BRCA1* PV, 2695 patients a *BRCA2* PV, and 35 patients both a *BRCA1* and *BRCA2* PV. For 461 patients, the type of *BRCA* PV was not specified. The reported incidence of STIC varied between the cohorts and ranged from 0 up to 10% of cases [20]. In a meta-analysis with random effect, a pooled estimated proportion of STIC of 2.8% (95% CI, 2.0–3.7) was found (Fig. 2).

**Table 1:** Characteristics of the included studies

Study	Year	Country	Setting	Retrospective/prospective	Number of patients		Number of STIC	% STIC
Carcangiu [21]	2004	Italy	Tertiary centre	Retrospective	26	2		7.69
Carcangiu [22]	2006	Italy	Tertiary centre	Retrospective	50	3		6.00
Lamb [23]	2006	USA	Tertiary centre	Prospective	62	4		6.45
Hirst [24]	2009	Australia	Multicentre	Retrospective	15	1		6.67
Rabban [25]	2009	USA	Tertiary centre	Retrospective	102	5		4.90
Shaw [26]	2009	Canada	Tertiary centre	Retrospective	176	15		8.52
Leonhardt [27]	2011	Germany	Tertiary centre	Retrospective	14	0		0.00
Manchanda [28]	2011	London, UK	Tertiary centre	Prospective	117	6		5.13
Powell [29]	2011	USA	Tertiary centre	Retrospective	111	5		4.50
Bacha [30]	2012	Canada	Tertiary centre	Retrospective	76	0		0.00
Mingels [31]	2012	Netherlands	Tertiary centre	Retrospective	226	14		6.19
Powell [32]	2013	USA	Multicentre	Retrospective	814	17		2.09
Reitsma [33]	2013	Netherlands	Tertiary centre	Prospective	303	3		0.99
Wethington [34]	2013	USA	Tertiary centre	Retrospective	375	10		2.67
Cass [20]	2014	USA	Tertiary centre	Retrospective	78	8		10.26
Conner [35]	2014	USA	Tertiary centre	Retrospective	302	5		1.66
Sherman [36]	2014	USA	Multicentre	Prospective	557	4		0.72
Malmberg [37]	2016	Sweden	Tertiary centre	Retrospective	42	1		2.38
Poon [38]	2016	Australia	Tertiary centre	Retrospective	72	3		4.17
Zakhour [39]	2016	USA	Tertiary centre	Retrospective	246	9		3.66
Ayres [40]	2017	Australia	Tertiary centre	Prospective	12	0		0.00
Bogani [41]	2017	Italy	Tertiary centre	Prospective	57	2		3.51
Lee (1) [42]	2017	Australia	Multicentre	Prospective	128	2		1.56
Lee (2) [43]	2017	Korea	Tertiary centre	Retrospective	36	2		5.56
Ricciardi [44]	2017	Italy	Tertiary centre	Prospective	276	7		2.54
Artioli [45]	2018	Italy	Regional centre	Retrospective	10	1		10.00
Minig [46]	2018	Spain	Multicentre	Retrospective	354	3		0.85
Thompson [47]	2018	Ireland	Tertiary centre	Retrospective	46	0		0.00
Vd Hoeven [48]	2018	Netherlands	Tertiary centre	Retrospective	235	2		0.85
Visvanathan [49]	2018	USA	Multicentre	Retrospective	366	12		3.28
Wong [50]	2018	USA	Tertiary centre	Retrospective	197	3		1.52
Blok [51]	2019	Netherlands	Tertiary centre	Retrospective	527	4		0.76
Rudaitis [52]	2019	Lithuania	Tertiary centre	Prospective	71	7		9.86
Stanciu [17]	2019	UK	Tertiary centre	Retrospective	244	6		2.46
Stewart [53]	2019	USA	Tertiary centre	Retrospective	61	3		4.92
Wilhite [54]	2019	USA	Multicentre	Retrospective	290	7		2.41
Cheng [55]	2020	China	Tertiary centre	Retrospective	24	1		4.17
Gornjec [56]	2020	Slovenia	Tertiary centre	Retrospective	145	3		2.07
Rush [57]	2020	USA	Tertiary centre	Prospective	371	8		2.16

Other aberrant epithelial lesions are reported on to a limited extent. The term “hyperplasia” is mentioned in three studies with an estimated incidence of 17.3% (95% CI, 0.0–94.5) [20, 31, 33]. “Atypia” is reported in five studies, with an estimated incidence of 11.0% (95% CI, 2.9–23.3) [20, 21, 28, 31, 33]. P53 signatures are reported on by six studies, with an estimated incidence of 16.2% (95% CI, 2.2–39.7) [16, 20, 25, 27, 40, 47]. Finally, STIL was reported on by six studies and has an estimated incidence of 1.6% (95% CI, 0.3–3.8) [16, 26, 27, 44, 47, 55].

### Totally embedding and SEE-FIM protocol

Out of the 39 studies examined, 20 studies report the consistent use of the SEE-FIM grossing protocol. Fourteen studies report that SEE-FIM was not (always) used, and five studies do not report on the use of a specific grossing protocol. The incidence of STIC in studies applying the SEE-FIM protocol was 2.8% (95% CI, 1.9–3.9), while an incidence of 2.7% (95% CI, 1.3–4.6) was found in studies without the SEE-FIM protocol (*p* = 0.92) (supplement Fig. 3).

**Fig. 3:**
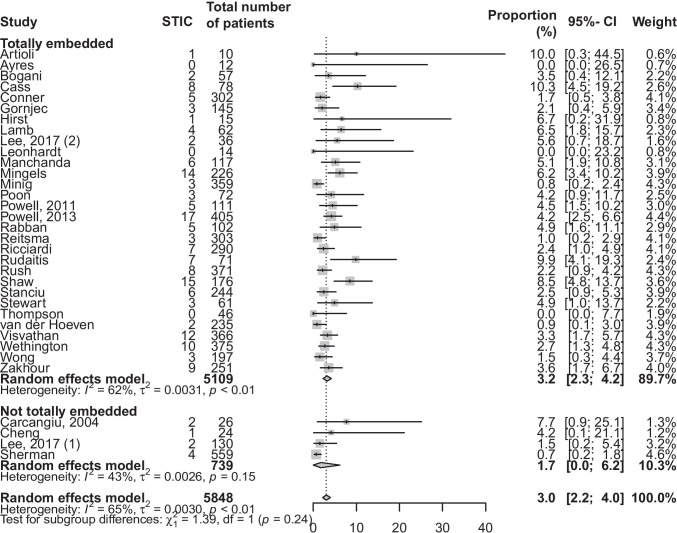
Forest plot representing the proportion of STIC, with subgroup analysis based on whether all specimens were totally embedded

Most of the studies did totally embed the risk reducing salpingo-oophorectomy specimens. Apart from the 20 studies who applied the SEE-FIM protocol, an additional 10 studies describe fully embedding all the specimens. This means that the entire fallopian tubes were embedded, but contrary to the SEE-FIM protocol, the fimbriated end was not sectioned parallel to the long axis of the fallopian tube. Out of the remaining nine studies, four studies report not always fully embedding the specimens. For the other five studies, this information was missing. When comparing the group of studies who totally embedded all specimens with the studies who did not, a respective difference in incidence of 3.2% (95% CI, 2.3–4.2) and 1.7% (95% CI, 0.0–6.2) was found (*p* = 0.24) (Fig. 3).

### Morphological criteria

**Fig. 4:**
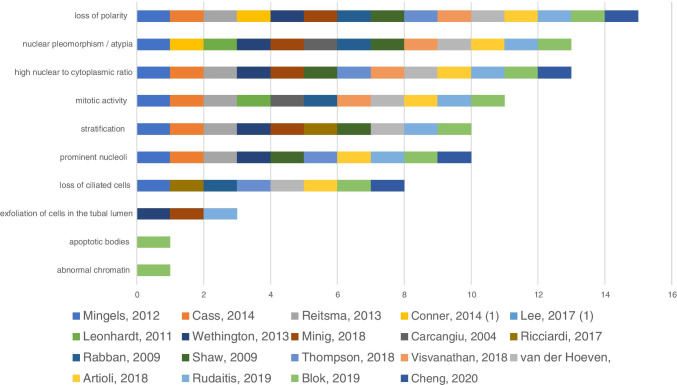
Morphological criteria attributed to STIC. The *x*-axis represents the number of studies reporting the criteria

A total of 19 studies described the morphological criteria used to identify potential STIC lesions. The most commonly mentioned characteristic was loss of polarity which was mentioned in 15 studies (15/19), followed by nuclear pleomorphism/atypia (13/19), high nuclear to cytoplasmic ratio (13/19), mitotic activity (11/19,) pseudostratification (10/19), prominent nucleoli (10/19), loss of ciliated cells (8 /19), detachment of cells from the surface (3/19), apoptotic bodies (1/19), and abnormal chromatin (1/19). The described morphological criteria used for identifying STIC are shown in Fig. 4.

### Subspecialized pathologist

Pathology specimens are reported to have been assessed by a subspecialized (or dedicated) gynecopathologist in 25 out of 39 studies. Of the remaining studies, one reported that a general pathologist analyzed the specimens, and 13 studies did not report on this feature. The studies with a reported subspecialized gynecopathologist had a STIC incidence of 3.1% (95% CI, 2.1–4.2), compared to an incidence of 2.3% (95% CI, 1.1–3.9) for the other studies (*p* = 0.34) (Supplement Fig. 4).

### Immunohistochemistry

The use of IHC in diagnosing STIC was described by 21 studies. All these 21 studies performed p53 stainings, and 19 studies used an additional Ki-67 marker. When we compared the studies describing the use of IHC, with those who did not, we saw an equal incidence of STIC in these groups of 2.8%. (95% CI for IHC group 1.6–4.2; 95% CI for unknown group 1.8–4.1). (Supplement Fig. 5). Ten of the studies also commented on the interpretation of IHC. Five studies considered an aberrant p53 staining pattern, either being overexpression or complete loss of expression, combined with an increased proliferative activity (Ki-67), a prerequisite for diagnosing STIC. The other 5 studies described IHC as being supportive, but not necessary for the diagnosis. A table, providing an overview on how these articles describe dealing with IHC, is provided in the supplementary appendix (supplement Fig. 6).

## Discussion

### Clinical relevance

It is essential to reliably diagnose or exclude STIC lesions. Firstly, because safety of novel preventive strategies in trial settings, such as salpingectomy with delayed oophorectomy, for women at high inherited risk for ovarian cancer, depends on STIC assessment. Secondly, because STIC at risk-reducing surgery is associated with increased risk to develop peritoneal carcinomatosis and might indicate for additional staging surgery and treatment, which is currently being debated. In this systematic review and meta-analysis, we provide a framework for diagnosing STIC. We analyzed the use of grossing protocols, the most commonly used morphologic criteria, the training level of pathologists, and the use of IHC.

### Grossing protocols

STIC diagnosis starts with a comprehensive grossing protocol. In our meta-analysis, we found a slightly higher detection of STIC when the specimens were fully embedded compared to studies who applied representative sampling. The additional value of the SEE-FIM protocol was not readily apparent in the data, yet makes theoretical sense. STIC is a lesion which is not macroscopically visible. Especially in the setting of risk reducing salpingo-oophorectomy, the lesions can be very small, ranging between < 1 and 11 mm [21, 22, 24, 25, 29, 39, 50]. Therefore, extensive sampling is vital. In addition, it is found that STIC often presents in the fimbriated end of the fallopian tube [19, 58]. In order to optimize the exposure of the distal fallopian tube, the SEE-FIM protocol was developed, which uses longitudinal sectioning of the fimbriated end [19]. The value of this protocol versus representative sampling in the setting of HGSC was demonstrated by Koc et al. They compared the outcomes of 39 cases of HGSC, examined according to the SEE-FIM protocol, with 113 cases, examined by representative sampling. In the SEE-FIM group, they found 15 STIC lesions, compared to 1 STIC lesion in the classic grossing method [59]. The SEE-FIM protocol could also be of added value in gyneco-oncological indication, other than risk reducing salpingo-oophorectomy or HGSC. For example, the International Society of Gynecological Pathologists now recommends to also use the SEE-FIM protocol in patients with endometrial carcinoma, or at least to include the entire fimbrial end [60].

### Morphologic criteria

The morphological criteria used in detecting STIC were mentioned by 16 out of the 32 studies we reviewed. The six most frequently mentioned criteria were (1) loss of polarity, (2) nuclear pleomorphism/atypia, (3) high nuclear to cytoplasmic ratio, (4) mitotic activity, (5) pseudostratification, and (6) prominent nucleoli. These criteria were all mentioned by more than half of the studies commenting on morphology. Whether these criteria were equally weighted and if these criteria are a prerequisite for diagnosing STIC remains unclear in these articles.

Fallopian tube tissue consists of stromal components which are lined by a predominantly single layer of secretory, ciliated, and intercalated cells. Recognizing epithelium as being aberrant is a cornerstone in the diagnostic process of STIC. However, standardized morphological criteria for STIC are lacking. Moreover, inter observer variability for recognizing aberrant fallopian tube epithelium is found to be high in multiple studies. Carlson et al. asked six pathologists and six pathology trainees to review a total of 30 cases, containing 14 STIC lesions. The majority agreed on 9 out of these 14 cases, leading to a minimal reproducibility, with a kappa (*k*) score of 0.333 [61]. Visvanathan also only found a weak reproducibility when assessment of STIC was based on morphology alone and found a *k*-score of 0.39 amongst five pathologists [62]. One can imagine that new technological developments in pathology, such as the use of deep learning algorithms in digitalized H&E slides, may eventually help in this task. Artificial intelligence algorithms have already shown to be able to perform tasks such as fully automated detection of breast cancer metastases in lymph nodes, and automated Gleason grading of prostate biopsies on the level of a subspecialized pathologist [63, 64]. However, for the time being, it is important for both pathologists and clinicians to at least be aware that there is a considerable degree of subjectivity to the morphological interpretation and pathologists are encouraged to seek a second opinion in case of doubt.

### Training level of the pathologist

A slightly higher number of STIC lesions were picked up in studies that explicitly mention that a subspecialized gynecopathologist performed the assessment. It must however be noted that a number of the articles did not clearly comment on whether specimens were seen by a general pathologist or a subspecialized gynecopathologist. One can imagine that a pathologist who works in a centre where there is a research interest in STIC will tend to have an above average expertise on the subject. The true skill level at an average hospital setting, where STIC might only be infrequently encountered in a risk reducing salpingo-oophorectomy setting, therefore remains unclear.

The importance of proper training was demonstrated in the difference between trainees and pathologist in Carlson’s study, whereby the agreement between experienced pathologist (*k* = 0.453) was better than that amongst trainees (*k* = 0.253) [61]. In addition to the importance of proper training in a general sense, we might ask what the value of a subspecialized pathologist would be in diagnosing STIC. The debate on sub specialization in pathology remains ongoing, whereby the practical downsides must be weighed against the benefits of expanded knowledge and experience [65, 66]. Even though many examinations of fallopian tube specimens could be considered routine work, specialist knowledge and experience may be needed to adequately recognize the special cases.

### Immunohistochemistry

Additional IHC staining is often used in diagnosing STIC, most notably p53 and Ki-67. Twenty-one studies mention the use of IHC; however, the remaining studies often did not comment on this. No difference in STIC incidence was found between the group that describes the use of IHC and the one that does not. However, this does not necessarily mean that IHC would not be of added value. IHC stainings might influence the incidence in two ways. On the one hand, IHC may lead to downgrading of cases, e.g., from STIC to STIL. On the other hand, more lesions might be identified due to increased sensitivity. As a result, a comparable incidence may be found regardless of the use of IHC.

How these stains are interpreted, either being a prerequisite or a supportive tool, often remains unclear in the articles. Though these stains can indeed prove helpful, we must be aware of how to interpret these stains. Previously suggested diagnostic algorithms often strongly rely on IHC. For example, Visvanathan et al. developed a model based on a combination of morphological suspicion of STIC and the results of p53 and Ki-67 stains [62]. Lesions would subsequently be classified as STIC, STIL, p53 signature, or reactive, based on the combination of these results. An alternative approach was proposed by Meserve et al. who presented a decision tree, starting at identifying altered epithelium and in subsequent steps checking for the presence of cilia, p53 immunostaining pattern, polarity of cells, and finally atypia [67]. The strict application of IHC in these algorithms was already debated by Perrone et al. [68]. They argued that if a lesion is morphologically unequivocally STIC, additional IHC staining can actually be confusing, and that the use of IHC should be reserved for indeterminate cases [68]. Algorithms such as the ones mentioned above can provide a welcome guidance in the diagnostic process, and also seem to improve reproducibility of the diagnosis [69]. There is no harm in using a low-threshold approach for ordering IHC stains, but one should be able to properly interpret these findings and not automatically reject a diagnosis of STIC in case of non-conclusive IHC results.

The tumour suppressor gene *TP53* has been shown to be mutated in approximately half of all human cancers, in 96.7% of HGSC and in approximately 92% of STIC, tested with *TP53* sequencing [10, 70, 71]. In the study from Kuhn et al., only exons 2–9 were sequenced, which will usually be sufficient; however, the actual percentage of STIC, harbouring a p53 mutation, may be higher. Molecular testing for *TP53* mutations is the gold standard. However, this is costly, labour intensive, and not always available. IHC can therefore indeed be an attractive substitute for molecular testing. The p53 stain is usually considered to be aberrant, when more than 75% of the nuclei, in a region of at least 12 epithelial cells, show an increased expression, or if there is an complete absence of staining [26, 62]. Kuhn et al. compared IHC staining with genetic testing and found a sensitivity of 87% and a specificity of 100% for IHC. Missense mutations were thereby associated with an overexpression in IHC, and the majority of truncating mutations showed complete loss of staining [7]. Kobel et al. also looked at the accuracy of IHC as a surrogate marker. They tested four different IHC assays for p53 and used next-generation sequencing as gold standard, to test 171 cases of HGSC. The best IHC assay thereby had a sensitivity of 96% and a specificity of 100% [72]. This indicates that IHC may have a high negative predictive value, but there will be a number of false negative cases, varying between 4 and 13%. It is important for the pathologist to be aware that IHC can give false negative results.

Additionally, Ki-67 can also play a supportive role in diagnosing STIC; however, the extent to which it can help to distinguish STIC from STIL or reactive lesions remains debatable. Ki-67 is a non-specific proliferation marker. An increased proliferative activity is usually defined as an overall Ki-67 expression of more than 10% of cells, but alternative systems, whereby the maximum proliferative index within a part of the lesion is taken, have also been proposed [12, 69] One of the challenges with Ki-67 however is the high risk of interlaboratory variability. Polley et al. compared staining of 100 breast tissue samples in eight different labs and concluded that cut-off values for Ki-67 for clinical decision making cannot be automatically transferred between laboratories [73].

### Strength and limitations

The strength of this review lies in the large number of inclusions, with 39 studies, accounting for a total of 6833 patients. To our knowledge, it is also the first time that these diagnostic features have all been considered in one review. The limitations of this study are found in the predominantly retrospective nature of these studies and the moderate heterogeneity [74]. Varying approaches and insights over time make comparisons of the outcomes of these studies suboptimal. Next to that, the low incidence of STIC and the moderate heterogeneity between studies contribute to insufficient discriminating power. Despite of these limitations, we feel that the collected data provides the best possible current overview on how to approach STIC diagnosis. Further standardization of the diagnostic approach will assist in stronger assessments and research in the future.

## Conclusion

**Fig. 5:**
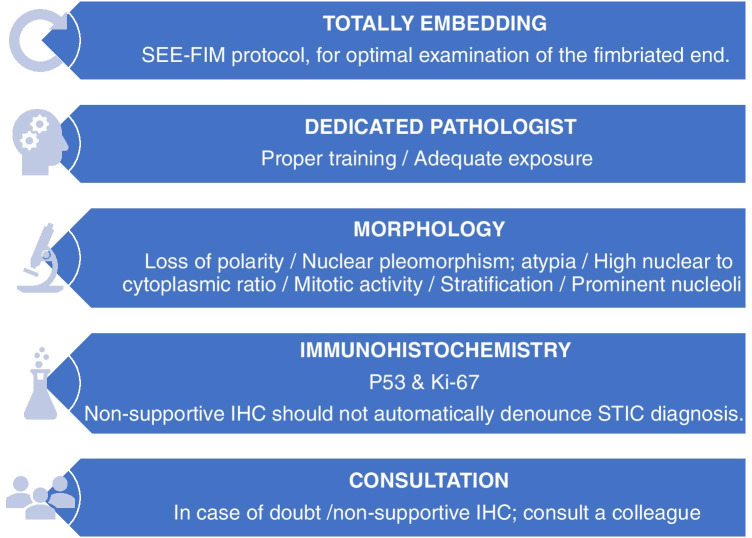
Framework for diagnosing STIC

Accurate and reproducible STIC diagnosis is important, both for individual patient care and for better understanding the oncogenesis of HGSC, but remains a challenging task. The diagnostic process can be broken down into several steps, which are highlighted in Fig. 5.

We believe that a good grossing protocol, evaluation by a subspecialized pathologist, rational use of immunohistochemical staining, and a low threshold for consultation with a colleague are the building blocks for a proper diagnostic approach toward STIC. Pathologists and clinicians alike should thereby be aware of the sliding scale in various other aberrant lesions, such as STIL and p53 signatures, and the challenges that occur when classifying them. Further standardization of the morphological criteria of STIC, a common approach in the diagnosis of other aberrant lesions in the fallopian tube and a better understanding of their clinical implications is needed.

## Supplementary Information

Below is the link to the electronic supplementary material.

Supplementary file1 (PDF 806 KB)

## Data Availability

The datasets used and analyzed during the current study are available from the corresponding author on reasonable request.
