# Supplementary material for: Recommendations for diagnosing STIC: a systematic review and meta-analysis
Source: Virchows Arch. 2021 Dec 1;480(4):725–37. doi: 10.1007/s00428-021-03244-w (PMC9023413; doi:10.1007/s00428-021-03244-w)
Supplement: Supplementary file 1 — Supplementary file1 (PDF 806 KB) [file 428_2021_3244_MOESM1_ESM.pdf]

SUPPLEMENTARY APPENDIX

Recommendations for diagnosing STIC. A review and meta-analysis.

Virchows Archiv

Authors: J.M.A. Bogaerts, M. P. Steenbeek, M.H.D. van Bommel, J. Bulten, J.A.W.M. van der Laak, J.A. de Hullu, M. Simons.

Corresponding author: J.M.A. Bogaerts, department of Pathology, Radboud University Medical Center, Nijmegen, The Netherlands. E-Mail: joep.bogaerts@radboudumc.nl

List of online supplementary Information:

- Supplement figure 1: PRISMA flow diagram on the selection of studies.
- Supplement figure 2: assessment for the risk of bias, using the checklist for observational Cohort and Cross-sectional studies of the National Heart, Lung and Blood institute (NIH).
- Supplement figure 3: forest plot representing the proportion of STIC, with subgroup analysis based on whether all specimens were examined according to the SEE-FIM protocol.
- Supplement figure 4: forest plot representing the proportion of STIC, with subgroup analysis based on whether studies mention a dedicated gynecopathologist.
- Supplement figure 5: forest plot representing the proportion of STIC, with subgroup analysis based on the reported use of IHC.
- Supplement figure 6: table describing the use of immunohistochemical stains.
- Search protocol.

SUPPLEMENTARY APPENDIX

Supplement figure 1: PRISMA flow diagram on the selection of studies.

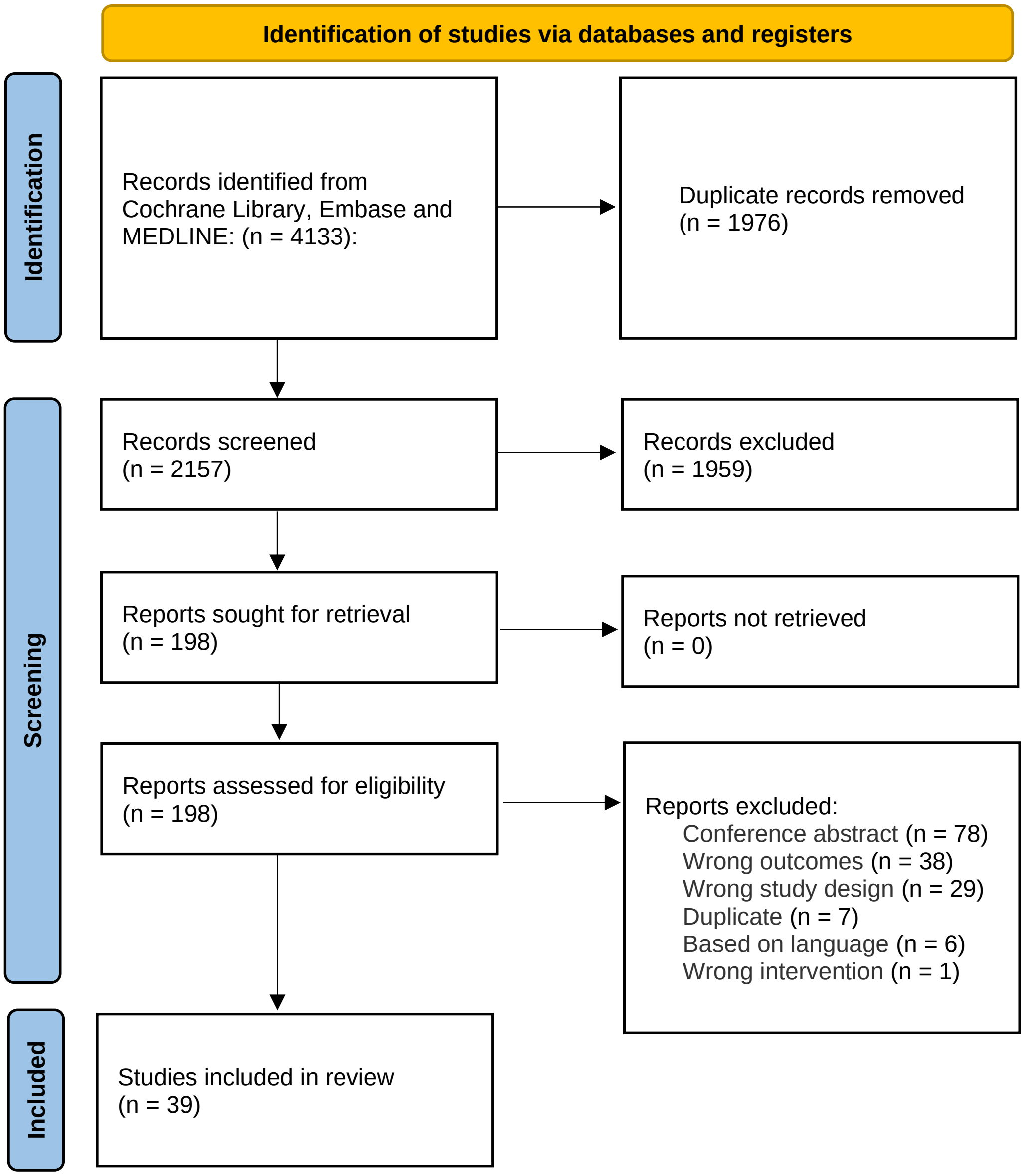

Supplement figure 2: assessment for the risk of bias, using the checklist for observational Cohort and Cross-sectional studies of the National Heart, Lung and Blood institute (NIH)

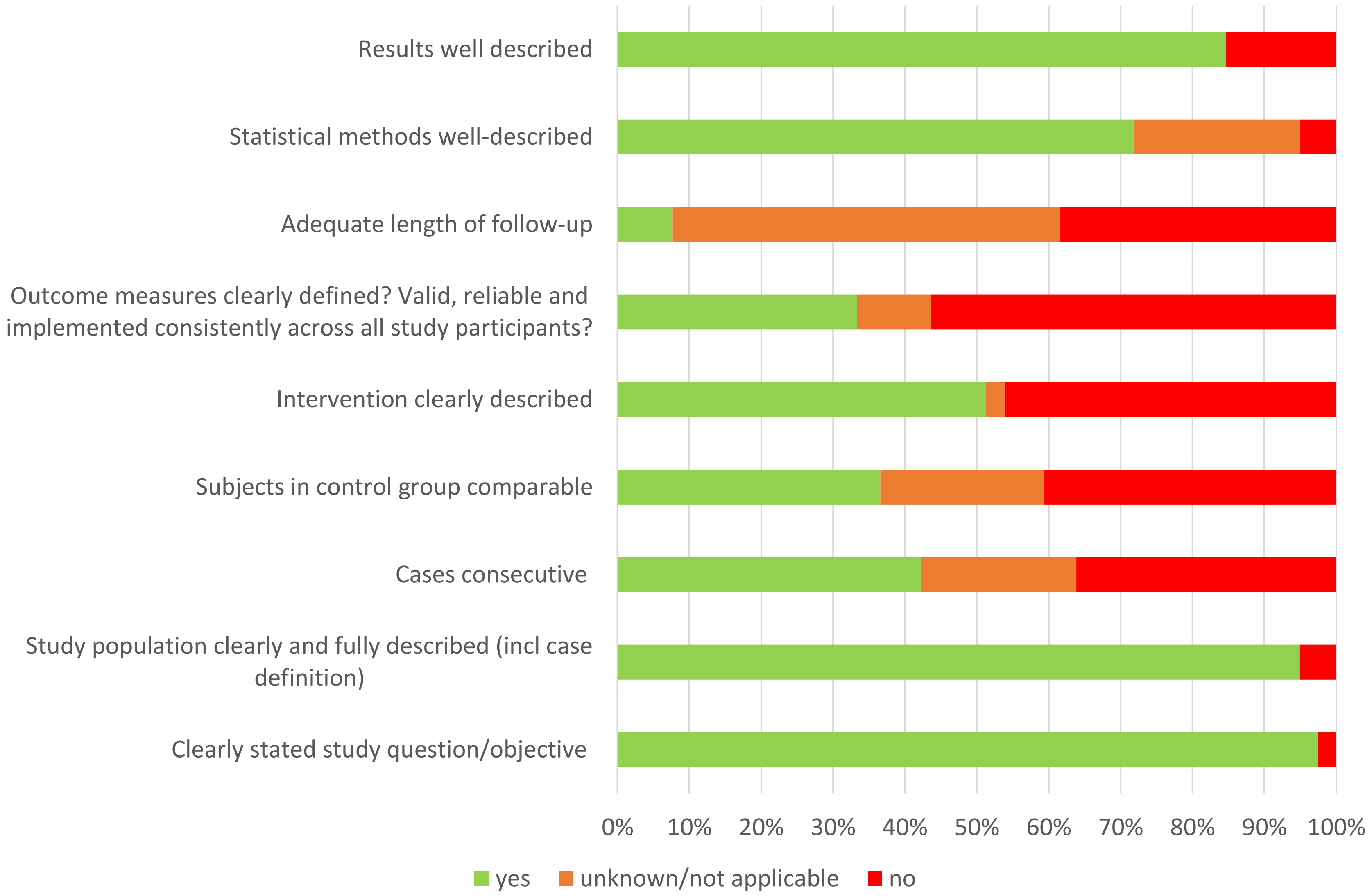

Supplement figure 3: Forest plot representing the proportion of STIC, with subgroup analysis based on whether all specimens were examined according to the SEE-FIM protocol.

Study	STIC	Total number of patients	Proportion (%)	95%−CI	Weight
**SEE FIM**					
Artioli	1	10	10.0	[0.3; 44.5]	0.6%
Ayres	0	12	0.0	[0.0; 26.5]	0.7%
Conner	5	302	1.7	[0.5; 3.8]	4.1%
Gornjec	3	145	2.1	[0.4; 5.9]	3.4%
Hirst	1	15	6.7	[0.2; 31.9]	0.8%
Lee, 2017 (2)	2	36	5.6	[0.7; 18.7]	1.7%
Mingels	14	226	6.2	[3.4; 10.2]	3.9%
Minig	3	359	0.8	[0.2; 2.4]	4.3%
Poon	3	72	4.2	[0.9; 11.7]	2.5%
Rabban	5	102	4.9	[1.6; 11.1]	3.0%
Reitsma	3	303	1.0	[0.2; 2.9]	4.1%
Ricciardi	7	290	2.4	[1.0; 4.9]	4.1%
Rudaitis	7	71	9.9	[4.1; 19.3]	2.5%
Stanciu	6	244	2.5	[0.9; 5.3]	3.9%
Stewart	3	61	4.9	[1.0; 13.7]	2.3%
Thompson	0	46	0.0	[0.0; 7.7]	1.9%
Visvathan	12	366	3.3	[1.7; 5.7]	4.3%
Wethington	10	375	2.7	[1.3; 4.8]	4.3%
Wilhite	7	290	2.4	[1.0; 4.9]	4.1%
Zakhour	9	251	3.6	[1.7; 6.7]	4.0%
**Random effects model**		**3576**	**2.8**	**[1.9; 3.9]**	**60.5%**
Heterogeneity: $I^2 = 53\%$, $\tau^2 = 0.0022$, $p < 0.01$					
**no SEE FIM**					
Bacha	0	76	0.0	[0.0; 4.7]	2.6%
Blok	4	527	0.8	[0.2; 1.9]	4.5%
Bogani	2	57	3.5	[0.4; 12.1]	2.2%
Carcangiu, 2004	2	26	7.7	[0.9; 25.1]	1.3%
Carcangiu, 2006	3	50	6.0	[1.3; 16.5]	2.0%
Cheng	1	24	4.2	[0.1; 21.1]	1.2%
Lamb	4	62	6.5	[1.8; 15.7]	2.3%
Lee, 2017 (1)	2	130	1.5	[0.2; 5.4]	3.3%
Malmberg	1	42	2.4	[0.1; 12.6]	1.8%
Manchanda	6	117	5.1	[1.9; 10.8]	3.1%
Powell, 2011	5	111	4.5	[1.5; 10.2]	3.1%
Shaw	15	176	8.5	[4.8; 13.7]	3.6%
Sherman	4	559	0.7	[0.2; 1.8]	4.6%
van der Hoeven	2	235	0.9	[0.1; 3.0]	3.9%
**Random effects model**		**2192**	**2.7**	**[1.3; 4.6]**	**39.5%**
Heterogeneity: $I^2 = 75\%$, $\tau^2 = 0.0050$, $p < 0.01$					
**Random effects model**		**5768**	**2.7**	**[1.9; 3.7]**	**100.0%**
Heterogeneity: $I^2 = 66\%$, $\tau^2 = 0.0032$, $p < 0.01$					
Test for subgroup differences: $\chi^2_1 = 0.01$, df = 1 ($p = 0.92$)					

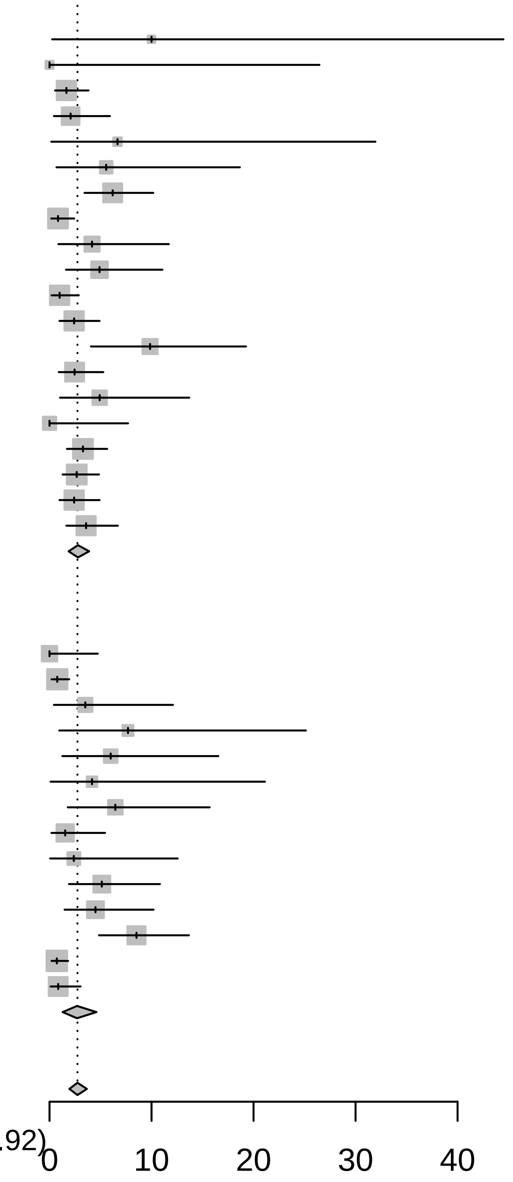

Supplement figure 4: Forest plot representing the proportion of STIC, with subgroup analysis based on whether studies mention a dedicated gynecopathologist.

Study	STIC	Total number of patients	Proportion (%)	95%−CI	Weight
**Dedicated gynecopathologist**					
Artioli	1	10	10.0	[0.3; 44.5]	0.5%
Ayres	0	12	0.0	[0.0; 26.5]	0.6%
Blok	4	527	0.8	[0.2; 1.9]	3.9%
Bogani	2	57	3.5	[0.4; 12.1]	1.9%
Cass	8	78	10.3	[4.5; 19.2]	2.3%
Hirst	1	15	6.7	[0.2; 31.9]	0.7%
Lamb	4	62	6.5	[1.8; 15.7]	2.0%
Lee, 2017 (2)	2	36	5.6	[0.7; 18.7]	1.5%
Manchanda	6	117	5.1	[1.9; 10.8]	2.7%
Mingels	14	226	6.2	[3.4; 10.2]	3.3%
Minig	3	359	0.8	[0.2; 2.4]	3.7%
Poon	3	72	4.2	[0.9; 11.7]	2.2%
Powell, 2013	17	405	4.2	[2.5; 6.6]	3.7%
Powell, 2011	5	111	4.5	[1.5; 10.2]	2.7%
Rabban	5	102	4.9	[1.6; 11.1]	2.6%
Reitsma	3	303	1.0	[0.2; 2.9]	3.5%
Ricciardi	7	290	2.4	[1.0; 4.9]	3.5%
Rush	8	371	2.2	[0.9; 4.2]	3.7%
Shaw	15	176	8.5	[4.8; 13.7]	3.1%
Sherman	4	559	0.7	[0.2; 1.8]	3.9%
Stanciu	6	244	2.5	[0.9; 5.3]	3.4%
Thompson	0	46	0.0	[0.0; 7.7]	1.7%
Visvathan	12	366	3.3	[1.7; 5.7]	3.7%
Wethington	10	375	2.7	[1.3; 4.8]	3.7%
Zakhour	9	251	3.6	[1.7; 6.7]	3.4%
**Random effects model**		**5170**	**3.1**	**[2.1; 4.2]**	**67.9%**
Heterogeneity: $I^2 = 72\%$, $\tau^2 = 0.0034$, $p < 0.01$					
**No dedicated gynecopathologist described**					
Bacha	0	76	0.0	[0.0; 4.7]	2.2%
Carcangiu, 2004	2	26	7.7	[0.9; 25.1]	1.2%
Carcangiu, 2006	3	50	6.0	[1.3; 16.5]	1.8%
Cheng	1	24	4.2	[0.1; 21.1]	1.1%
Conner	5	302	1.7	[0.5; 3.8]	3.5%
Gornjec	3	145	2.1	[0.4; 5.9]	2.9%
Lee, 2017 (1)	2	130	1.5	[0.2; 5.4]	2.8%
Leonhardt	0	14	0.0	[0.0; 23.2]	0.7%
Malmberg	1	42	2.4	[0.1; 12.6]	1.6%
Rudaitis	7	71	9.9	[4.1; 19.3]	2.2%
Stewart	3	61	4.9	[1.0; 13.7]	2.0%
van der Hoeven	2	235	0.9	[0.1; 3.0]	3.4%
Wilhite	7	290	2.4	[1.0; 4.9]	3.5%
Wong	3	197	1.5	[0.3; 4.4]	3.2%
**Random effects model**		**1663**	**2.3**	**[1.1; 3.9]**	**32.1%**
Heterogeneity: $I^2 = 49\%$, $\tau^2 = 0.0035$, $p = 0.02$					
**Random effects model**		**6833**	**2.8**	**[2.0; 3.7]**	**100.0%**
Heterogeneity: $I^2 = 66\%$, $\tau^2 = 0.0034$, $p < 0.01$					
Test for subgroup differences: $\chi^2_1 = 0.90$, df = 1 ($p = 0.34$)					

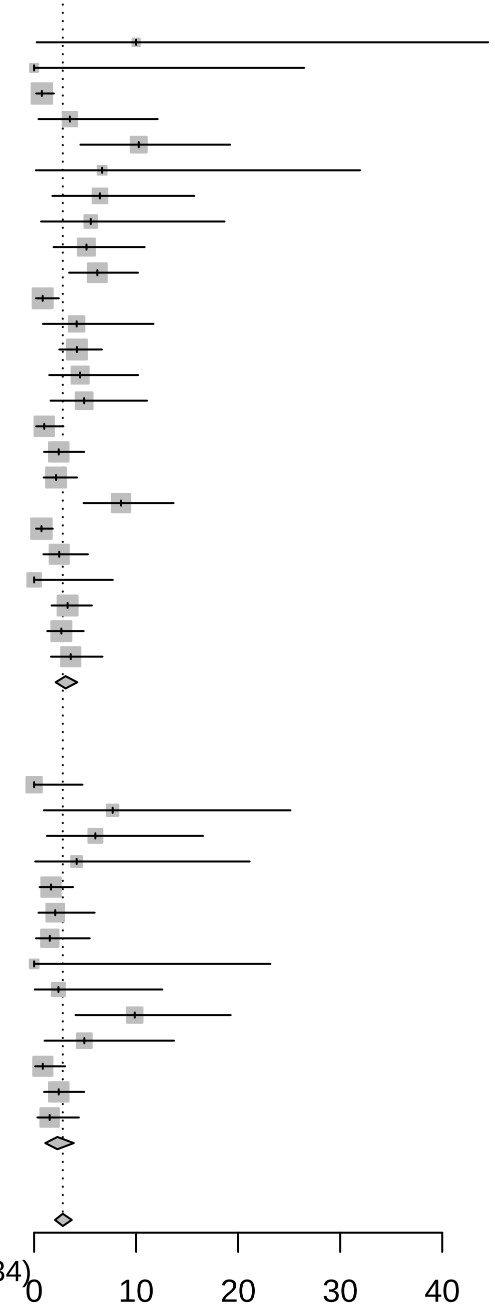

Supplement figure 5: Forest plot representing the proportion of STIC, with subgroup analysis based on the reported use of IHC.

Study	STIC	Total number of patients	Proportion (%)	95%−CI	Weight
**No IHC described**					
Artioli	1	10	10.0	[0.3; 44.5]	0.5%
Bacha	0	76	0.0	[0.0; 4.7]	2.2%
Bogani	2	57	3.5	[0.4; 12.1]	1.9%
Carcangiu, 2006	3	50	6.0	[1.3; 16.5]	1.8%
Gornjec	3	145	2.1	[0.4; 5.9]	2.9%
Hirst	1	15	6.7	[0.2; 31.9]	0.7%
Lamb	4	62	6.5	[1.8; 15.7]	2.0%
Manchanda	6	117	5.1	[1.9; 10.8]	2.7%
Mingels	14	226	6.2	[3.4; 10.2]	3.3%
Powell, 2013	17	405	4.2	[2.5; 6.6]	3.7%
Powell, 2011	5	111	4.5	[1.5; 10.2]	2.7%
Rush	8	371	2.2	[0.9; 4.2]	3.7%
Sherman	4	559	0.7	[0.2; 1.8]	3.9%
Stewart	3	61	4.9	[1.0; 13.7]	2.0%
van der Hoeven	2	235	0.9	[0.1; 3.0]	3.4%
Wilhite	7	290	2.4	[1.0; 4.9]	3.5%
Wong	3	197	1.5	[0.3; 4.4]	3.2%
Zakhour	9	251	3.6	[1.7; 6.7]	3.4%
**Random effects model**		**3238**	**2.8**	**[1.8; 4.1]**	**47.7%**
Heterogeneity: $I^2 = 65\%$, $\tau^2 = 0.0026$, $p < 0.01$					
**IHC**					
Ayres	0	12	0.0	[0.0; 26.5]	0.6%
Blok	4	527	0.8	[0.2; 1.9]	3.9%
Carcangiu, 2004	2	26	7.7	[0.9; 25.1]	1.2%
Cass	8	78	10.3	[4.5; 19.2]	2.3%
Cheng	1	24	4.2	[0.1; 21.1]	1.1%
Conner	5	302	1.7	[0.5; 3.8]	3.5%
Lee, 2017 (1)	2	130	1.5	[0.2; 5.4]	2.8%
Lee, 2017 (2)	2	36	5.6	[0.7; 18.7]	1.5%
Leonhardt	0	14	0.0	[0.0; 23.2]	0.7%
Malmberg	1	42	2.4	[0.1; 12.6]	1.6%
Minig	3	359	0.8	[0.2; 2.4]	3.7%
Poon	3	72	4.2	[0.9; 11.7]	2.2%
Rabban	5	102	4.9	[1.6; 11.1]	2.6%
Reitsma	3	303	1.0	[0.2; 2.9]	3.5%
Ricciardi	7	290	2.4	[1.0; 4.9]	3.5%
Rudaitis	7	71	9.9	[4.1; 19.3]	2.2%
Shaw	15	176	8.5	[4.8; 13.7]	3.1%
Stanciu	6	244	2.5	[0.9; 5.3]	3.4%
Thompson	0	46	0.0	[0.0; 7.7]	1.7%
Visvathan	12	366	3.3	[1.7; 5.7]	3.7%
Wethington	10	375	2.7	[1.3; 4.8]	3.7%
**Random effects model**		**3595**	**2.8**	**[1.6; 4.2]**	**52.3%**
Heterogeneity: $I^2 = 69\%$, $\tau^2 = 0.0046$, $p < 0.01$					
**Random effects model**		**6833**	**2.8**	**[2.0; 3.7]**	**100.0%**
Heterogeneity: $I^2 = 66\%$, $\tau^2 = 0.0034$, $p < 0.01$					
Test for subgroup differences: $\chi^2_1 = 0.01$, df = 1 ($p = 0.93$)					

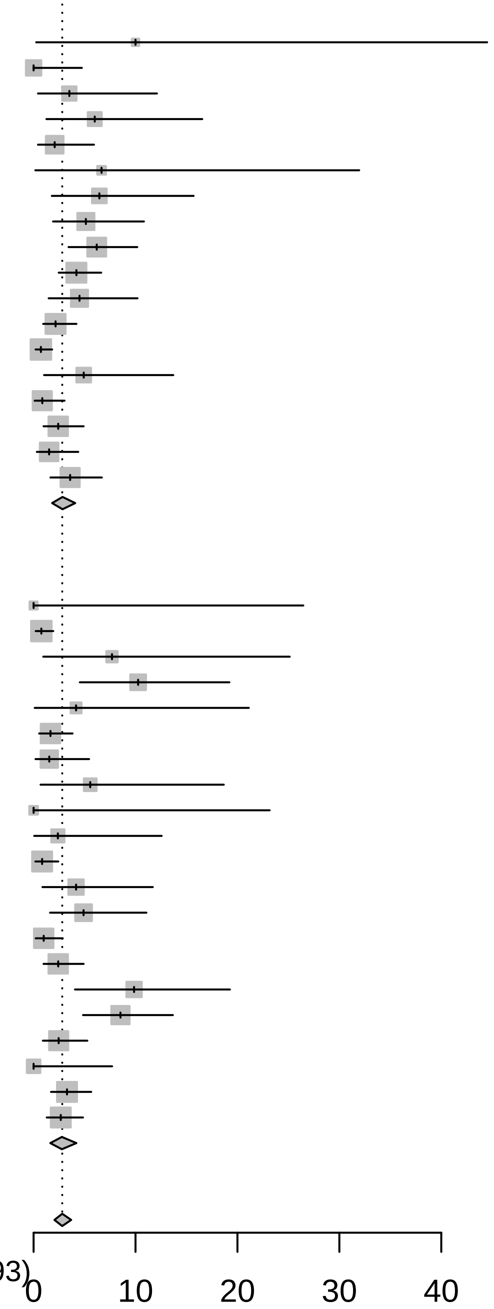

Supplement figure 6: table describing the use of immunohistochemical stains.

Author	Year	Aim of the study	P53	Ki67	Description of use of IHC
Shaw	2009	Validate previous findings in a larger series from BRCA1/2 mutation carriers, and further characterize precursors by histological assessment and IHC.	uncertain *	necessary	All fallopian tube sections were stained for p53 and Ki67. p53 was considered positive if greater than 75% of nuclei stained positive in a region exceeding 12 cells. Ki67 was considered increased if there was nuclear staining in a discrete focus greater than twice that of adjacent epithelium.<br><br>* Only describes P53 overexpression pattern. Null pattern or cytoplasmic pattern are not mentioned. The value of P53 in this setting is difficult to compare to current knowledge.
Leonhardt	2011	Evaluate the role of the fimbriated end of fallopian tubes with regard to p53 signature, TILT, and STIC in cases of different kinds of serous pelvic cancer.	necessary	necessary	p53 signature: p53 accumulation in 12 or more consecutive secretory cell nuclei, with very low proliferative index and no cytologic atypia; TILT: intermediate lesions between p53 signature and STIC, with a low/moderate proliferative index, no cytologic atypia, but p53 accumulation; STIC: composed of secretory cells showing a high proliferative index, significant atypia, architectural alterations, and strong staining for p53.
Wethington	2013	Identify isolated STIC and assess the clinical outcomes of these cases.	supportive	supportive	IHC was performed only when nuclear atypia was present. IHC stains included p53 and Mib-1. Elevated Mib-1 (>15% nuclear cell staining) and abnormal p53 staining (null phenotype or >60% nuclear cell staining) were used as supportive evidence of the diagnosis.
Conner	2014	Compute the risk of clinically silent adnexal neoplasia in women with germline BRCA1/2 mutations.	supportive	supportive	Histologic criteria of HGTIN consist of a combination of marked nuclear atypia and some loss of cell polarity, typically accompanied by an increased proliferative index and either strong or absent immuno-positivity for p53.
Lee	2017	Determine the quality of RRSO surgery and pathology	necessary	necessary	Based on morphology, and immunostaining for p53 and Ki67 as recommended by Visvanathan et al.
Minig	2018	Determine the incidence of STIC in BRCA mutation carriers after RRSO, as well as to describe oncological outcomes after RRSO.	supportive	supportive	Elevated Mib-1 (>15%) and abnormal p53 staining (null phenotype or > 60%) were used as supportive evidence of the diagnosis. Following the recommendation of the College of American Pathologists, IHC stains was not necessary in the presence of STIC, but if there was diagnostic uncertainty, both p53 and MIB-1 staining were performed
Thompson	2018	Analysis of indications and outcomes of RRSO	necessary	necessary	STIC is diagnosed based on a combination of atypical morphology, aberrant immunohistochemical expression of P53 (mutation type pattern of either strong diffuse expression or absent staining) and an increased proliferation rate.
Visvanathan	2018	Determine the prevalence of STIC and STIL and identify novel epidemiologic and clinical risk/protective factors associated with these precursor lesions.	necessary	necessary	p53 was scored as aberrant if diffuse expression (>75% of the cell) was present in at least 12 epithelial cells or there was complete absence of staining or nonabnormal pattern, and Ki-67 was categorized as <10% or >10% staining. STICs are expected to be laminin g1 positive, p53 diffuse, or completely negative and Ki-67 > 10%.
Blok	2019	Determine the prevalence of high grade serous carcinoma and STIC in BRCA1/2 carriers presenting for RRSO, and their follow-up.	supportive	supportive	IHC with p53 and MIB-1 was conducted for the cases with a HGSC and/or STIC. Diffuse intense staining with p53 was noted as 'mutation pattern', and complete absence of staining was noted as 'null-pattern'. Mutation or null-pattern p53 staining with a MIB-1 labeling index ≥10% were considered confirmatory for the diagnosis of STIC
Cheng	2020	To evaluate the benefit of RRSO by estimating the pathological positive rate of occult lesions, including STIC and occult cancers.	supportive	supportive	The diagnosis of STIC is based on a combination of morphological features. In addition, IHC features supporting the diagnosis of STIC include p53 status (overexpression >60% or no expression) and increased proliferative activity as reflected by the Ki-67 index.

Search protocol:

EMBASE search

	# ▲	Searches	Results	Type
☐	1	BRCA1 protein/	14846	Advanced
☐	2	BRCA2 protein/	10767	Advanced
☐	3	exp "hereditary breast and ovarian cancer syndrome"/	442	Advanced
☐	4	BRCA*.ti,ab,kw.	26044	Advanced
☐	5	((Hereditary or familial or high risk) adj3 ovar* adj3 (cancer* or carcinoma* or neoplas*)).ti,ab,kw.	3154	Advanced
☐	6	1 or 2 or 3 or 4 or 5	32062	Advanced
☐	7	exp Salpingectomy/	3937	Advanced
☐	8	salpingectom*.ti,ab,kw.	2824	Advanced
☐	9	Fallopian Tubes/	1195	Advanced
☐	10	exp ovariectomy/	34664	Advanced
☐	11	(RRBSO or BSO or oophorectom* or RRSO or ovariectom* or ((risk reduc* or prophylactic) adj2 ovar*)).ti,ab,kw.	47251	Advanced
☐	12	7 or 8 or 9 or 10 or 11	61239	Advanced
☐	13	6 and 12	2020	Advanced
☐	14	uterine tube tumor/	1334	Advanced
☐	15	epithelium hyperplasia/	2353	Advanced
☐	16	Pathology/	796006	Advanced
☐	17	histology/	627732	Advanced
☐	18	STIC.ti,ab,kw.	779	Advanced

19	Tubal intraepithelial carcinoma*.ti,ab,kw.	371	Advanced
20	(ovar* and (cancer or carcinoma)).ti,ab,kw.	111520	Advanced
21	patholog*.ti,ab,kw.	1008566	Advanced
22	fallopian tube*.ti,ab,kw.	12604	Advanced
23	hyperplasia.ti,ab,kw.	113244	Advanced
24	atypia.ti,ab,kw.	15131	Advanced
25	14 or 15 or 16 or 17 or 18 or 19 or 20 or 21 or 22 or 23 or 24	2355446	Advanced
26	13 and 25	1550	Advanced

MEDLINE search

# ▲	Searches	Results	Type
1	genes, BRCA1/	5588	Advanced
2	genes, BRCA2/	3551	Advanced
3	BRCA1 protein/	4961	Advanced
4	BRCA2 protein/	3520	Advanced
5	"Hereditary Breast and Ovarian Cancer Syndrome"/	205	Advanced
6	BRCA*.ti,ab,kf.	16614	Advanced
7	((Hereditary or familial or high risk) adj3 ovar* adj3 (cancer* or carcinoma* or neoplas*)).ti,ab,kf.	2121	Advanced
8	1 or 2 or 3 or 4 or 5 or 6 or 7	18817	Advanced
9	exp salpingectomy/ or salpingo-oophorectomy/	1079	Advanced
10	salpingectom*.ti,ab,kf.	1781	Advanced
11	Fallopian Tubes/su [Surgery]	3233	Advanced
12	(RRBSO or BSO or oophorectom* or RRSO or ovariectom* or ((risk reduc* or prophylactic) adj2 ovar*)).ti,ab,kf.	37665	Advanced

☐	13	exp ovariectomy/ or salpingo-oophorectomy/	23970	Advanced
☐	14	fallopian tube neoplasms/	2700	Advanced
☐	15	9 or 10 or 11 or 12 or 13	48104	Advanced
☐	16	8 and 15	1060	Advanced
☐	17	Hyperplasia/	32070	Advanced
☐	18	STIC.ti,ab,kf.	434	Advanced
☐	19	Tubal intraepithelial carcinoma*.ti,ab,kf.	186	Advanced
☐	20	Epithelial ovarian cancer.ti,ab,kf.	8103	Advanced
☐	21	(ovar* and (cancer or carcinoma)).ti,ab,kf.	77497	Advanced
☐	22	patholog*.ti,ab,kf.	736770	Advanced
☐	23	fallopian tube*.ti,ab,kf.	10432	Advanced
☐	24	hyperplasia.ti,ab,kf.	84257	Advanced
☐	25	atypia.ti,ab,kf.	10070	Advanced
☐	26	14 or 17 or 18 or 19 or 20 or 21 or 22 or 23 or 24 or 25	907384	Advanced
☐	27	16 and 26	823	Advanced

Cochrane search

Search Name: RRSO review_Cochrane search

ID Search Hits

#1 MeSH descriptor: [Genes, BRCA2] this term only 52

#2 MeSH descriptor: [Genes, BRCA1] this term only 66

#3 MeSH descriptor: [BRCA1 Protein] this term only 53

#4 MeSH descriptor: [BRCA2 Protein] this term only 40

#5 MeSH descriptor: [Hereditary Breast and Ovarian Cancer Syndrome] this term only 9

#6 (BRCA*):ti,ab,kw 777

#7 ((Hereditary or familial or high risk) AND ovar* AND (cancer* or carcinoma* or neoplas*)):ti,ab,kw 501

#8 #1 or #2 or #3 or #4 or #5 or #6 or #7 1179

#9 MeSH descriptor: [Salpingectomy] this term only 34

#10 MeSH descriptor: [Ovariectomy] this term only 272

#11 MeSH descriptor: [Salpingo-oophorectomy] this term only 0

#12 (salpingectom*):ti,ab,kw 186

#13 MeSH descriptor: [Fallopian Tubes] this term only 166

#14 (RRBSO or BSO or oophorectom* or RRSO or ovariectom* or ((risk reduc* or prophylactic) adj2 ovar*)):ti,ab,kw 890

#15 #9 or #10 or #11 or #12 or #13 or #14 1185

#16 #8 and #15 59

#17 MeSH descriptor: [Hyperplasia] this term only 500

#18 STIC:ti,ab,kw 22

#19 (Tubal intraepithelial carcinoma*):ti,ab,kw 1

#20 (ovar* and (cancer or carcinoma)):ti,ab,kw 5579

#21 (patholog*):ti,ab,kw 59326

#22 (fallopian tube*):ti,ab,kw 884

#23 (hyperplasia):ti,ab,kw. 4537

#24 (atypia):ti,ab,kw 237

#25 #17 or #18 or #19 or #20 or #21 or #22 or #23 or #24 68161

#26 #16 and #25 55
